# Supplementary material for: Lipid Droplet-Associated Proteins Perilipin 1 and 2: Molecular Markers of Steatosis and Microvesicular Steatotic Foci in Chronic Hepatitis C
Source: Int J Mol Sci. 2022 Dec 7;23(24):15456. doi: 10.3390/ijms232415456 (PMC9778710; doi:10.3390/ijms232415456)
Supplement: Supplementary file 1 [file ijms-23-15456-s001.zip › ijms-1986277-supplementary_submitted.pdf]

Table S1. Clinical and histological parameters of patients with chronic hepatitis B

Patient-ID (* = Biopsy with PLIN2-positive foci)	Grading (1-4) according to Desmet (81)	Staging (1-4) according to Desmet (81)	Age (years)	Sex (male = 1, female = 2)	HBV DNA viral load (IU/ml)
201	1	1	43	1	4,000
202*	2	2	23	1	140,000
203	1	1	45	1	70
204*	1	1	34	2	2,200
205*	1.5	2.5	27	1	690,000
206	1.5	1	27	2	180
207	0	0.5	34	1	580
208	1	1.5	66	2	1,700
209	1.5	1.5	48	1	1,600
210	1.5	1.5	27	1	340
211	2	4	42	1	570,000
212*	0	2	33	1	70
213	2	2	51	2	16,000
214	1.5	1.5	48	1	64,000
215	1	0	59	2	1,000
216	1	1	61	2	75,000
217	1	0	40	1	1,300
218	2	3.5	35	2	42,000,000
220	2.5	4	48	2	116
221	1	1	45	2	3,100
222	3	2	59	1	150,000,000
223	0.5	0	24	2	99,000
224*	1	0	37	2	76,000
225	1.5	0	34	2	22,000
226*	2	1	30	2	250
227	3	2	59	2	190,000
228	2	1.5	24	1	8,700
229	2	2	47	1	unknown
230	2.5	4	49	1	unknown
231	2	2	48	2	unknown
232	1	1	30	2	unknown
233	3	4	56	2	unknown
234	2	2	43	2	unknown

Abbreviations: DNA: deoxyribonucleic acid; HBV: hepatitis B virus; ID: identification number; PLIN2: perilipin 2

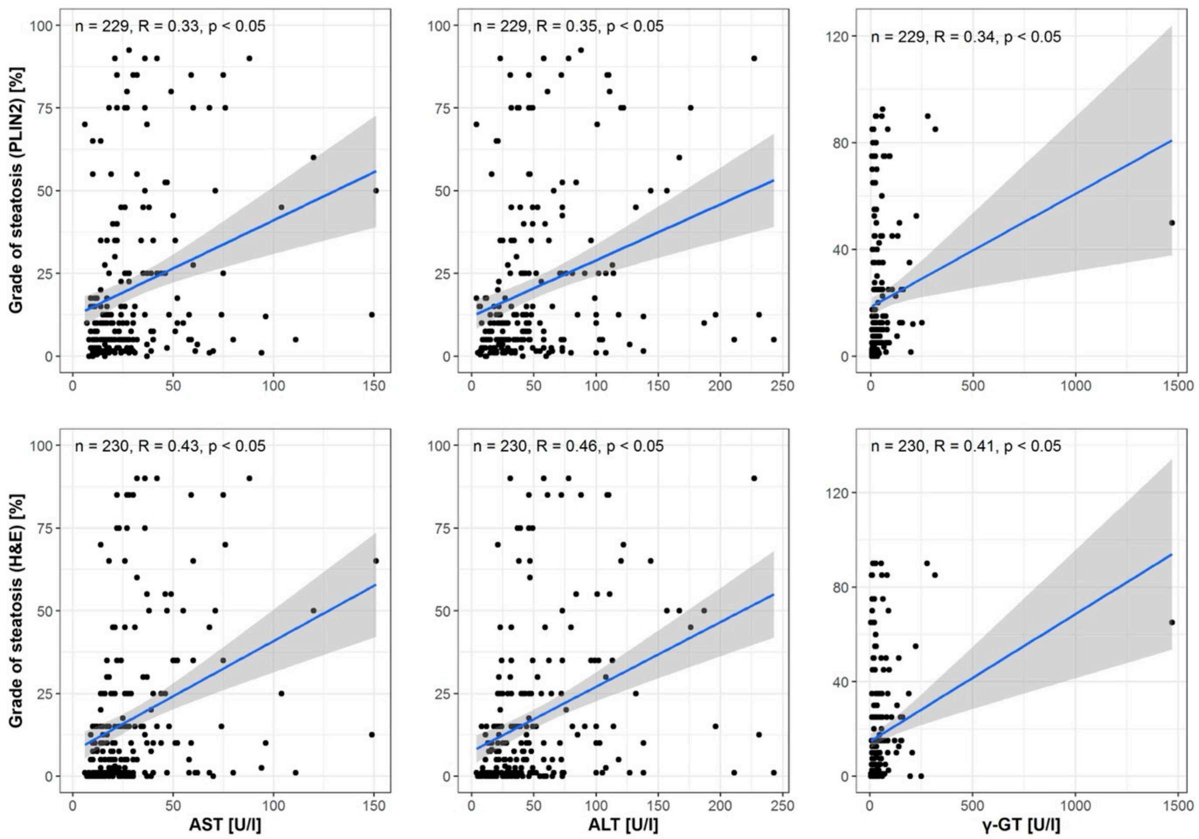

Figure S1. Correlation of steatosis with liver enzyme and gamma-GT levels in patients with chronic hepatitis C (Spearman). Steatosis grade was determined by H&E morphology or perilipin 2 immunohistochemistry.

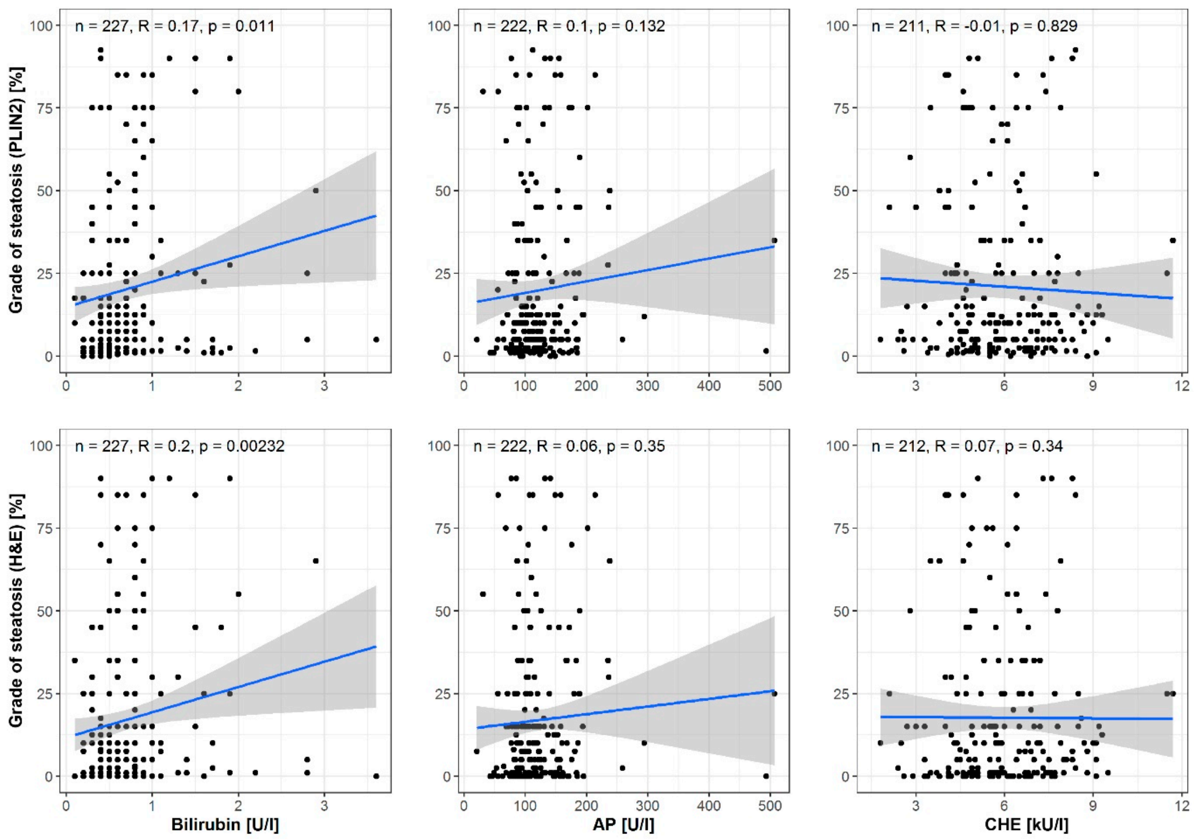

Figure S2. Correlation of steatosis with bilirubin, alkaline phosphatase and cholinesterase in patients with chronic hepatitis C (Spearman). Steatosis grade was determined by H&E morphology or perilipin 2 immunohistochemistry.

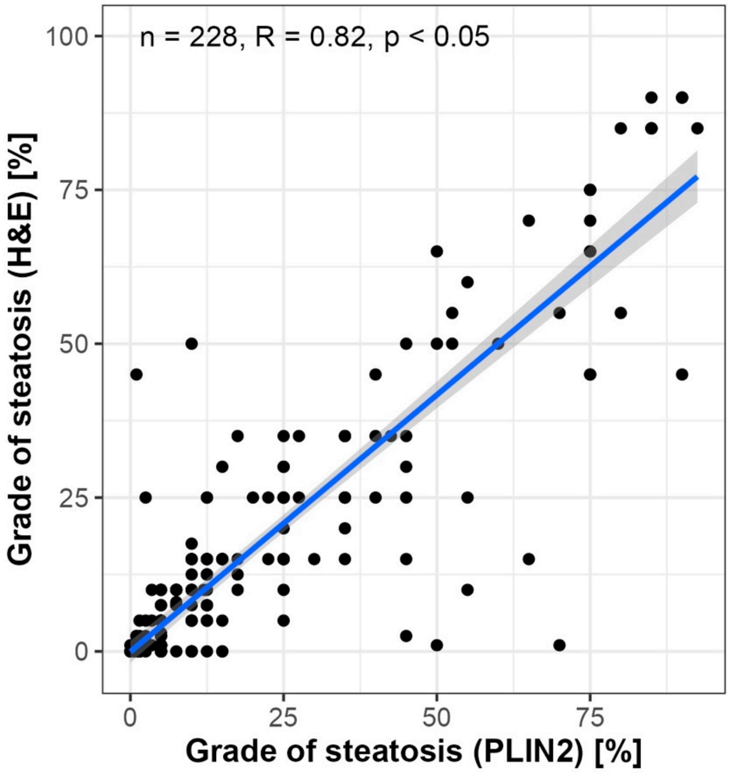

Figure S3. Correlation of steatosis determined by H&E morphology and steatosis determined by perilipin 2 immunohistochemistry in patients infected with chronic hepatitis C (Spearman analysis).

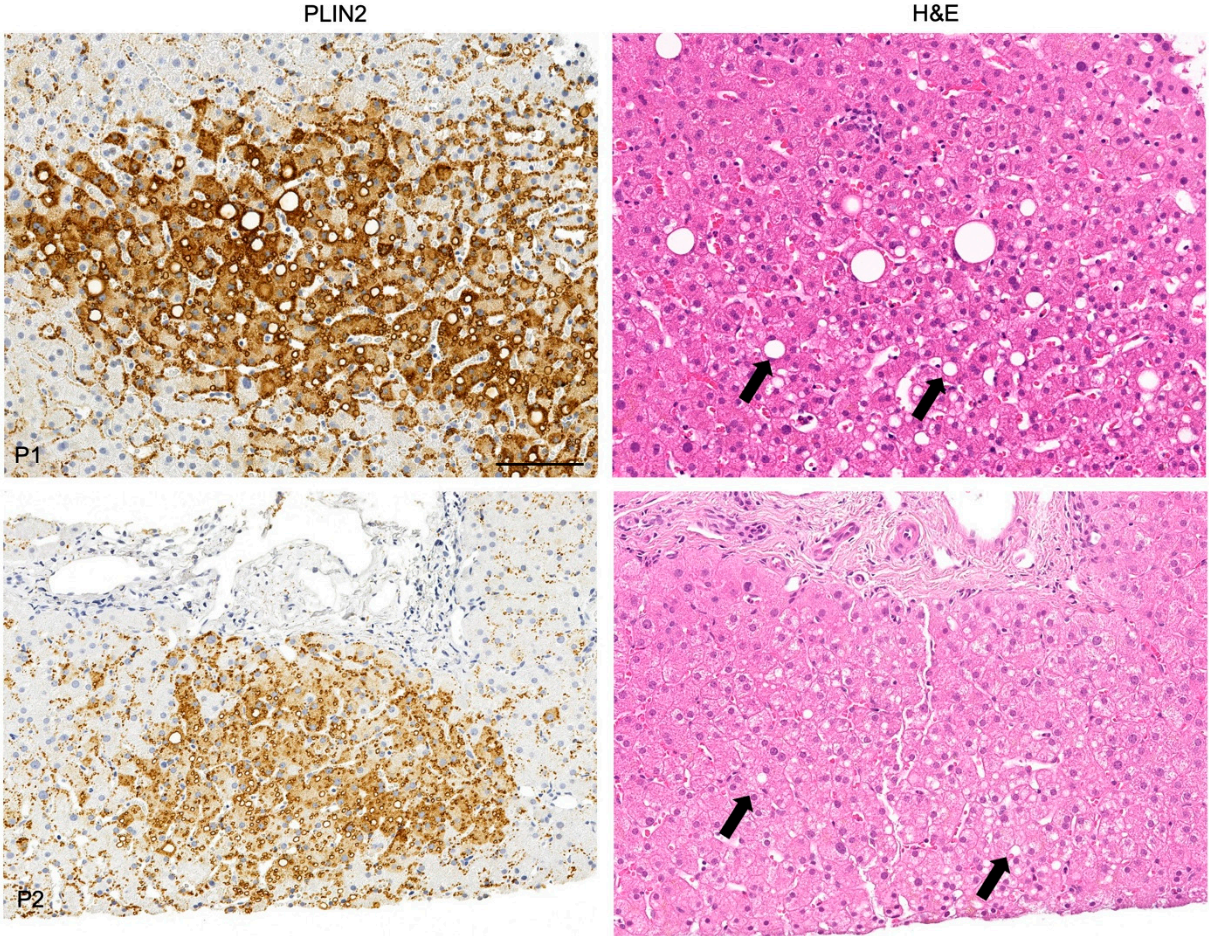

Figure S4. Immunohistochemical staining of liver biopsies from two patients (P1 and P2) with chronic hepatitis B and clearly definable perilipin 2-positive foci. Perilipin 2-positive foci could not be predicted in routine H&E staining. Only larger LDs were detectable in the H&E staining (arrows). Scale bar 100 µm.

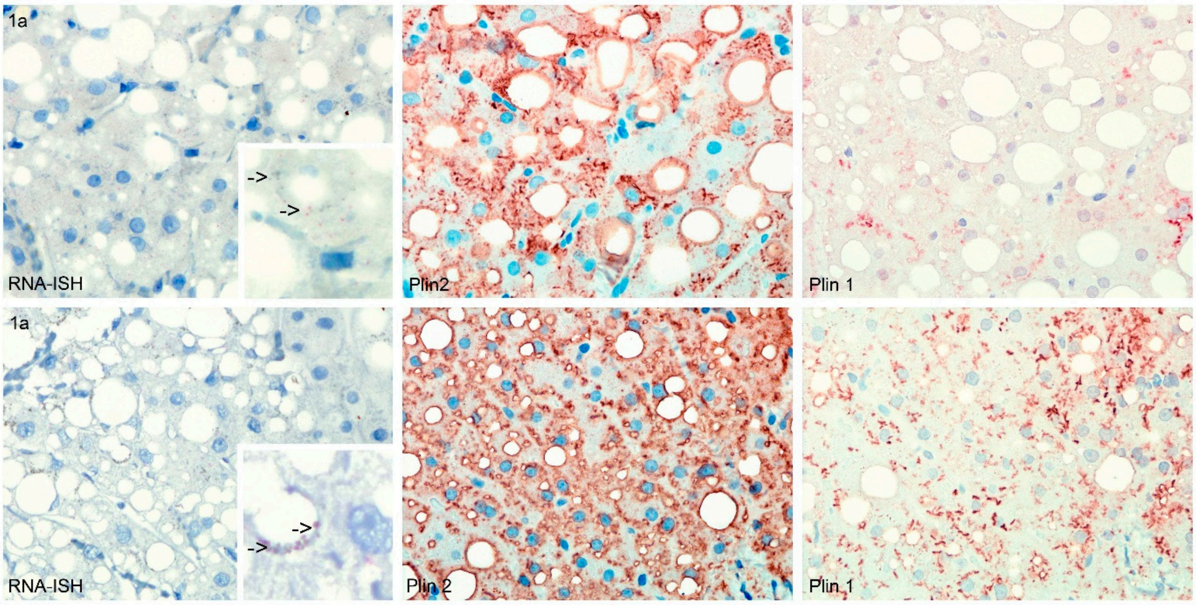

Figure S5. HCV RNA in situ hybridization in a liver biopsy of a patient with chronic hepatitis C, genotype 1a. Two areas depicted with each RNA CISH with perilipin 2 (plin2) and perilipin 1 (plin1) staining of the same area from consecutive sections. Magnification: 400x.

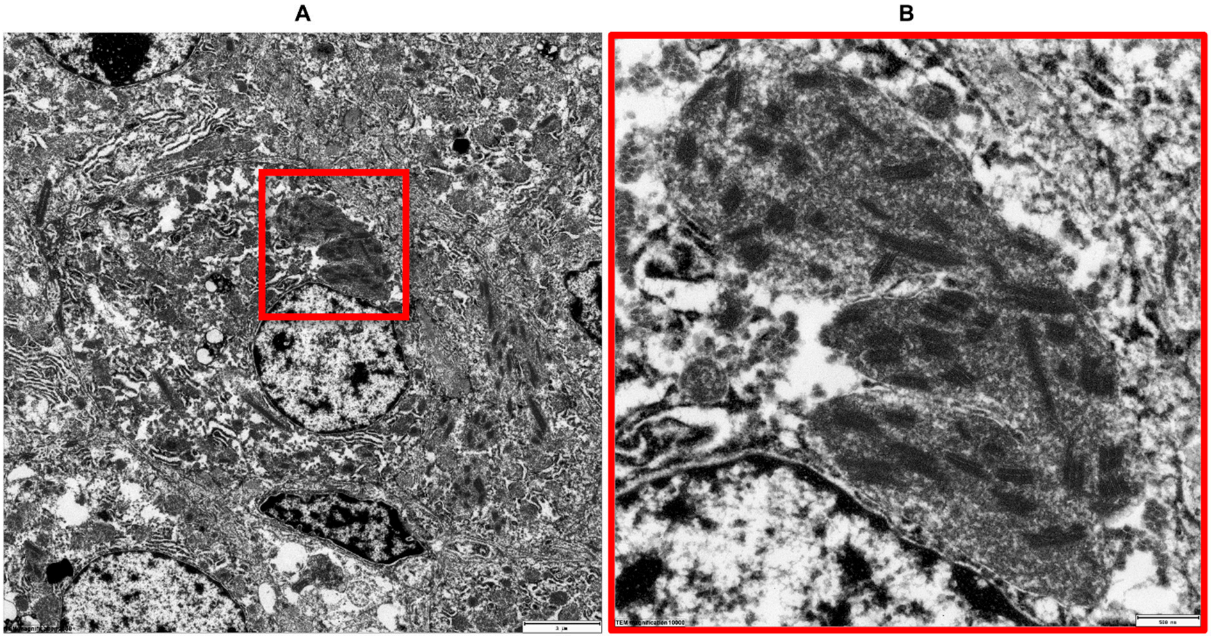

Figure S6. Transmission electron micrographs of non-neoplastic liver of one patient demonstrating atypical mitochondria with crystalline inclusions (B, enlargement of A). The patient received chemotherapy for the treatment of liver metastases of colorectal adenocarcinoma, which may result in these reactive mitochondrial abberations. In the hepatocyte shown in A, only small LDs are shown.

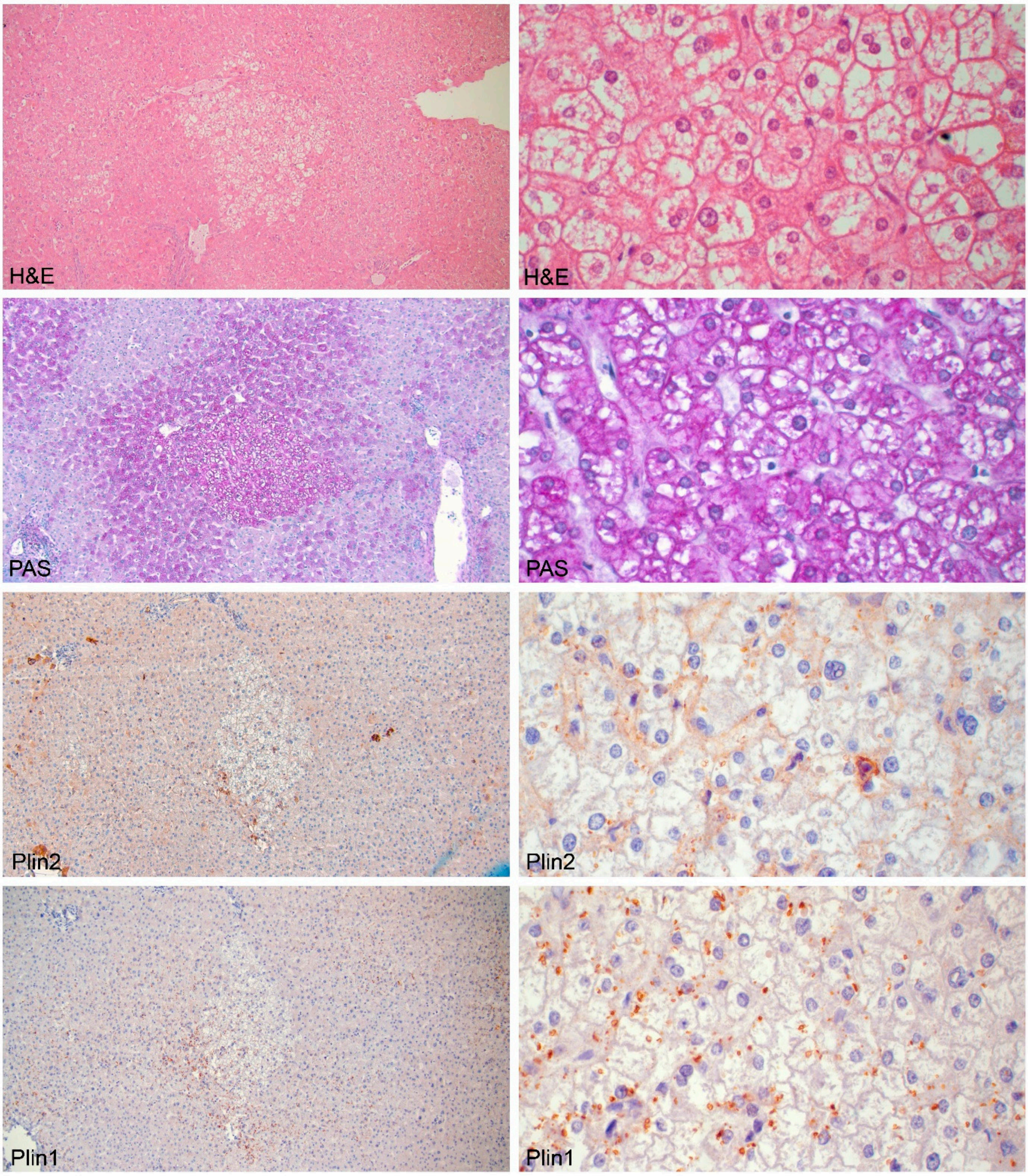

Figure S7. Clear cell foci do not overlap with perilipin-positive steatotic foci. Clear cell focus in a liver resection specimen of a 47 year old women with liver metastases of ovarian carcinoma. H&E and PAS-stain as well as perilipin 1 and perilipin 2 immunohistochemistry (Plin1, Plin2) on consecutive sections. Perilipin 1 and 2 staining decorate some small LDs in hepatocytes in the clear cell focus and surrounding liver, but no strong cytoplasmic staining is observed. Magnification: left side: 100x, right side: 600x.
